# Neonatal Microbiome, Intestinal Inflammation, and Necrotizing Enterocolitis

**DOI:** 10.3390/microorganisms10071382

**Published:** 2022-07-09

**Authors:** Kathryn Y. Burge, Troy A. Markel

**Affiliations:** 1 Department of Pediatrics, Division of Neonatal-Perinatal Medicine, University of Oklahoma Health Sciences Center, Oklahoma City, OK 73104, USA; 2 Department of Surgery, Section of Pediatric Surgery, Riley Hospital for Children at Indiana University Health, Indianapolis, IN 46202, USA; tmarkel@iupui.edu

## 1. Introduction

Necrotizing enterocolitis (NEC), the most common gastrointestinal emergency in the neonatal intensive care unit (NICU), is a leading cause of preterm infant morbidity and mortality. This multifactorial disease is heavily predicated on intestinal barrier immaturity, hyperinflammatory immune cells, and dysbiosis. Intestinal inflammation, often closely following enteral feeds, results in bacterial translocation of the intestinal barrier, sepsis, multiorgan failure, and, frequently, death [1]. Despite decades of research progress, NEC pathophysiology remains incompletely understood, and treatment options are limited to surgical and supportive therapies. Infants surviving this devastating disease often suffer from neurodevelopmental impairment, short gut syndrome, intestinal failure, and cerebral palsy [2].

Recently, animal modeling in germ-free or Toll-like receptor knockout (TLR KO) mice has indicated dysbiosis is likely required, but not sufficient, in driving NEC pathogenesis [3,4]. A number of factors, both modifiable and unmodifiable, affect gut microbiome development in preterm infants, including infant gestational age, mode of delivery and feeding, infant medications, and maternal and NICU environment. In this Special Issue, we highlight recent developments in understanding of preterm infant gut microbiome development and progression; present methods to noninvasively detect shifts in microbiome composition for use as biomarkers; and introduce promising developments in the use of prebiotics, probiotics, or synbiotics for prevention or amelioration of severe intestinal inflammation in premature infants.

## 2. Infant Gut Microbiota

The development of the infant microbiome is highly dynamic and easily influenced by host, maternal, and environmental factors. While research has implicated a number of pathogenic constituents of the NEC microbiome, interactions between host epithelial and immune cells, including alleles predisposing these infants to NEC risk, and the collective intestinal microbiome, likely dictate NEC pathogenesis. Compared with healthy term infants, the microbiome of preterm infants is characterized by distinct, but less diverse, taxa, with a progression dictated largely by postmenstrual age [5]. Components of these host–microbe interactions are implicated in basal tuning of the preterm immune system to luminal contents, breakdown of the intestinal barrier, and resulting runaway intestinal epithelial inflammation characteristic of NEC.

## 3. Microbiome Detection and Identification of Biomarkers

Volatile organic compounds (VOCs) have recently emerged as noninvasive fecal biomarkers of microbiome shifts, with the potential to diagnose NEC before obvious symptoms are present. VOCs are detectable through gas chromatography (GC), mass spectrometry (MS), and, very recently, electronic nose (eNose) technology. Use of eNose technology to detect NEC in animal models has been promising, but clinical applications have been limited due to non-standardized protocols for stool acquisition, as well as differences in signals among age groups and dietary patterns [6]. Metagenomic analysis taking into consideration functional enzymatic profiles of preterm microbial communities has indicated elevated DL-lactate, as well as a reduction in enzymes capable of utilizing human milk oligosaccharides (HMOs), may prove useful as biomarkers of developing preterm dysbiosis and NEC [7].

## 4. Prebiotics, Probiotics, and Synbiotics

Prebiotics (beneficial substrates of host microorganisms), probiotics (microorganisms with a demonstrated host benefit), and synbiotics (mixtures of pre- and probiotics) are of continuing interest for prevention or therapeutic use in NEC. These supplements, when used in the early postnatal period, are capable of modulating preterm infant microbiome development, thus potentially mitigating or preventing the development of NEC. Differences in study designs, enrollment criteria, and supplement composition have resulted in wide variability among randomized controlled trial (RCT) outcomes thus far, but more targeted study populations and higher standards for purity using commercially produced supplements are likely to show benefit in preterm infants at risk of developing NEC [8].

In this Special Issue, entitled “Neonatal Microbiome, Intestinal Inflammation, and Necrotizing Enterocolitis”, recent influences on, iterative improvements in, and challenges to healthy neonatal microbiome development, particularly in relation to intestinal inflammation and necrotizing enterocolitis, are reported.
